# Propofol TIVA vs. inhalational anesthesia for spine surgery: in‑hospital mortality and postoperative complications in a nationwide Korean cohort

**DOI:** 10.1186/s12871-025-03385-4

**Published:** 2025-10-17

**Authors:** Tak Kyu Oh, Saeyeon Kim, In-Ae Song

**Affiliations:** 1 Department of Anesthesiology and Pain Medicine, Seoul National University Bundang Hospita, Seongnam-si, South Korea; 2 Department of Anesthesiology and Pain Medicine, College of Medicine, Seoul National University, Seoul, South Korea; 3 Department of Anesthesiology and Pain Medicine, Seoul National University Bundang Hospital, Gumi-ro, 173, Beon-gil, Seongnam, 13620 South Korea

**Keywords:** General anesthesia, Spine surgery, Total intravenous anesthesia, Volatile inhalants

## Abstract

**Background:**

Given propofol’s antioxidant and anti‑inflammatory properties compared with volatile/inhalational agents, we aimed to evaluate the association between anesthetic technique and both in‑hospital mortality and postoperative complications following spinal surgery.

**Methods:**

In this retrospective, population‑based cohort study, we used South Korea’s National Health Insurance Service database to identify adult patients (≥ 18 years) who underwent spinal surgery between January 1, 2016 and December 31, 2021. Primary outcomes were in‑hospital mortality and postoperative complications. Propensity score (PS) matching (1:1) was employed to balance baseline characteristics between the total intravenous anesthesia (TIVA) and volatile/inhalational anesthesia (INH) groups.

**Results:**

Among 708,387 patients, 264,728 (37.4%) received TIVA and 443,659 (62.6%) received INH. After PS matching, 460,654 patients remained (230,327 per group). In the PS‑matched cohort, TIVA was associated with significantly lower odds of in‑hospital mortality (OR 0.85; 95% CI 0.80–0.89; *P* = 0.004) and postoperative complications (11.8% vs. 14.2%; OR 0.81; 95% CI 0.80–0.82; *P* < 0.001) compared with INH. In the full cohort, multivariable logistic regression confirmed these findings: TIVA remained linked to reduced in‑hospital mortality (OR 0.74; 95% CI 0.63–0.87; *P* < 0.001) and fewer postoperative complications (OR 0.71; 95% CI 0.70–0.73; *P* < 0.001).

**Conclusions:**

In this nationwide cohort, propofol‑based TIVA was associated with lower in‑hospital mortality and fewer postoperative complications than volatile/inhalational anesthesia in adult spinal surgery patients. Prospective trials are warranted to confirm these findings.

**Trial registration:**

: Not applicable.

**Supplementary Information:**

The online version contains supplementary material available at 10.1186/s12871-025-03385-4.

## Background

Spinal surgery is inherently invasive, often requiring extended operative times, additional neuromonitoring, and vigilant postoperative care to minimise morbidity and mortality. As global life expectancy rises and age‑standardized mortality declines, age‑related degenerative spinal disorders are increasingly common; elective lumbar fusion procedures in the United States rose by 62.3% from 2004 to 2015, and in‑hospital mortality among patients over 65 years remains approximately four times higher than in younger cohorts [1, 2]. With an overall postoperative complication rate of ~ 16.4% in spine surgery [3], the choice of anesthetic technique plays a critical role in modulating surgical stress responses and influencing patient outcomes.

Anesthetic modality can alter systemic inflammation, oxidative stress, and organ perfusion during and after surgery. Total intravenous anesthesia (TIVA) with propofol and opioids offers potent antioxidant and anti‑inflammatory effects, whereas volatile/inhalational agents (e.g., sevoflurane, desflurane, isoflurane) have distinct impacts on immune function, cytokine release, and microcirculatory dynamics [4–6]. In non‑spinal settings, retrospective studies have linked TIVA to lower postoperative complication rates and improved long‑term survival in cancer and cardiac surgery [7–9], although some analyses report minimal differences [10]. Despite these findings in other surgical fields, direct comparisons of propofol-based TIVA versus volatile/inhalational anesthesia specifically in spinal surgery are limited, and no large randomized controlled trials have been conducted in this surgical population.

To address this gap, we performed a nationwide, population‑based cohort study using South Korea’s National Health Insurance Service (NHIS) database, comparing in‑hospital mortality and postoperative complication rates between propofol‑based TIVA and volatile/inhalational anesthesia in adult spinal surgery patients. Our objective was to provide real‑world evidence to guide anesthetic selection in this high‑risk surgical population.

## Methods

### Study design and ethical statement

The Institutional Review Board approved this retrospective population-based cohort study (approval number: X-2304-821-901). The NHIS Big Data Centre granted permission for data sharing for this project (NHIS-2023-1-525). Because data analyses were done retrospectively and anonymised data were taken from the South Korean NHIS database, the requirement for informed consent was waived. The study was conducted in accordance with the Helsinki Declaration of 1975 (revised in 2008).

### Data source

The South Korean NHIS database, which included comprehensive details on all diagnoses, prescriptions, and medical treatments, provided the data used for this analysis. The International Classification of Diseases, Tenth Revision (ICD-10) codes were used to document diagnoses. The NHIS database also contains socioeconomic and demographic data about South Korean individuals.

### Inclusion/exclusion criteria

We identified adult patients (≥ 18 years) in the NHIS database who underwent spinal surgery under general anesthesia between January 1, 2016 and December 31, 2021, including cervical, thoracic, lumbar, and unspecified procedures (Table S1). To ensure each surgical episode represented an independent unit of analysis—and to avoid correlated outcomes within the same individual—we retained only the first eligible spinal surgery per patient and excluded all subsequent spine operations, representing 81,194 procedures (10.2% of the initial 789,581 cases). While this exclusion may omit a subgroup with higher comorbidity burdens or differing anesthetic exposures, it yields a more homogeneous sample in which each observation reflects a unique patient event. By enforcing this criterion, we upheld the independence assumption required for valid logistic regression modeling and minimized intra‑patient clustering bias.

### TIVA and inhalation anesthesia groups

The anesthetic method used for spine surgery was divided into TIVA and inhalation anesthesia (INH) groups. Patients who received an inhalational anaesthetic (e.g. sevoflurane, desflurane, or isoflurane) were assigned to the INH group, whereas patients who were continuously infused with propofol for anesthesia were assigned to the TIVA group. A patient was also assigned to the INH group if general anesthesia was maintained with inhalational anaesthetics and propofol was used once for induction. Other agents—nitrous oxide and etomidate—are not routinely used for maintenance in Korean spinal surgery and were not examined, and remimazolam was not yet available in Korea during the study period.

### Study endpoints

In-hospital mortality and postoperative complications were the two study endpoints. A death occurring while a patient was in hospital following spine surgery was referred to as in-hospital mortality. Postoperative complications were classified and extracted using ICD-10 codes during hospitalisation following spine surgeries. These included acute coronary events (I21, I22, and I252), heart failure (I50), pulmonary embolism (I26), acute and subacute hepatic failure (K720), acute kidney injury (N17), sepsis (A40 and A41), wound infection (T793 and T814), pneumonia (J12 to J18 and J69), urinary tract infection (N17), haemorrhage (D62, D68.3, J94.2, J95.00, O71.701, O71.704, O71.801, O72.00, O72.10, O72.20, O90.20, P12.0, S06.4, S06.5, S06.6, S27.100, S27.200, S27.300, S36.090, S36.091, S36.150, S36.151, S36.800, S36.810, S37.000, S37.300, T79.2, T81.0), and central nervous system complications (E11.0, E15, F05.0, F05.1, F05.8, F05.9, G00.3, G37.2, G97.2, O74.30, O89.40, S06.0, S06.1, S06.25, S06.35, S06.4, S06.5, S06.6, S06.85, S06.9). The prior report served as the basis for the categorisation criteria for postoperative complications [11, 12].

### Study parameters/Covariates

Age and sex information were obtained. Covariates for patient socioeconomic status were residence, employment status (including self-employed), and household income. One of five household income groups included medical aid programs and a four-quartile ratio. The government enrols low-income people who cannot pay insurance premiums in medical aid programs. Urban areas were the capital and other metropolitan cities; rural areas were the rest. We collected data on the level of spinal surgery and types of procedures performed. The types of spine surgeries were arthrodesis, corpectomy, spine fracture surgery, discectomy, spine tumour surgery, laminectomy, and other spine surgeries. In addition, we gathered information on the use of intraoperative neurophysiological monitoring (IONM).

The Charlson Comorbidity Index and underlying disability were applied to consider the coexisting conditions of the patients. ICD-10 codes added to the NHIS database were used to calculate the Charlson Comorbidity Index scores at the time of hospital admission (Table S1). All disabilities in South Korea must be registered in the NHIS database to be eligible for the different benefits provided by social welfare programs. Every disability needs to be formally identified by a medical expert who assesses the challenges encountered when performing regular activities. Table S2 provides a comprehensive classification of impairments. Depending on the severity of the conditions, the patients were placed into one of six severity categories (1st: most severe; 6th: least severe). Grades one through three were assigned a ‘severe’ level, and grades four through six were assigned a ‘mild to moderate’ level.

### Statistical analysis

Continuous variables are presented as means ± SD and categorical variables as counts (percentages). To minimize confounding, we performed 1:1 nearest‑neighbor propensity score (PS) matching (caliper 0.25, no replacement) [13]. This caliper choice follows the recommendation of Rosenbaum and Rubin, who—drawing on Cochran and Rubin’s simulation results—suggested matching on the log‑odds with a 0.25 SD caliper to achieve optimal bias reduction while preserving sample size [14]. PSs were estimated via logistic regression including prespecified covariates—namely age, sex, household income quartile, residence (urban/rural), employment status, Charlson Comorbidity Index, registered disability level, spinal level (cervical, thoracic, lumbar), procedure type (arthrodesis, corpectomy, spine fracture repair, discectomy, tumor surgery, laminectomy, other), and IONM use. The absolute standardised difference (ASD) was utilised to assess the balance between the TIVA and INH groups before and after PS matching. An ASD of < 0.1 was established to determine if the groups were well-balanced through PS matching. For categorical variables with k levels, we calculated ASDs for the k-1 non-reference categories only, omitting the reference level to avoid redundancy arising from linear dependence among the dummy indicators [15]. The PS-matched cohort was subjected to univariable logistic regression analysis to ascertain whether the TIVA group exhibited a distinct risk of in-hospital mortality or postoperative complications compared to the INH group. Results are presented as odds ratios (ORs) with 95% confidence intervals (CIs).

For sensitivity analyses, we built multivariable logistic regression models to evaluate whether the findings in the PS-matched sample were applicable to the overall cohort. This sensitivity analysis allowed us to adjust for the fact that PS matching discards a significant number of samples.

Prespecified subgroup analyses by spinal region (cervical, thoracic, lumbar), age group (≥ 75 years), and high comorbidity burden (Charlson Comorbidity Index ≥ 5) were performed. The Hosmer-Lemeshow statistic was used to assess the model’s goodness of fit. There were no concerns regarding multicollinearity among the variables, as all variance inflation factors were less than 2.0. The R software (version 4.0.3, R packages, R Project for Statistical Computing, Vienna, Austria) was used for all statistical analyses, with *P* < 0.05 denoting statistical significance.

## Results

### Patient

**Fig. 1:**
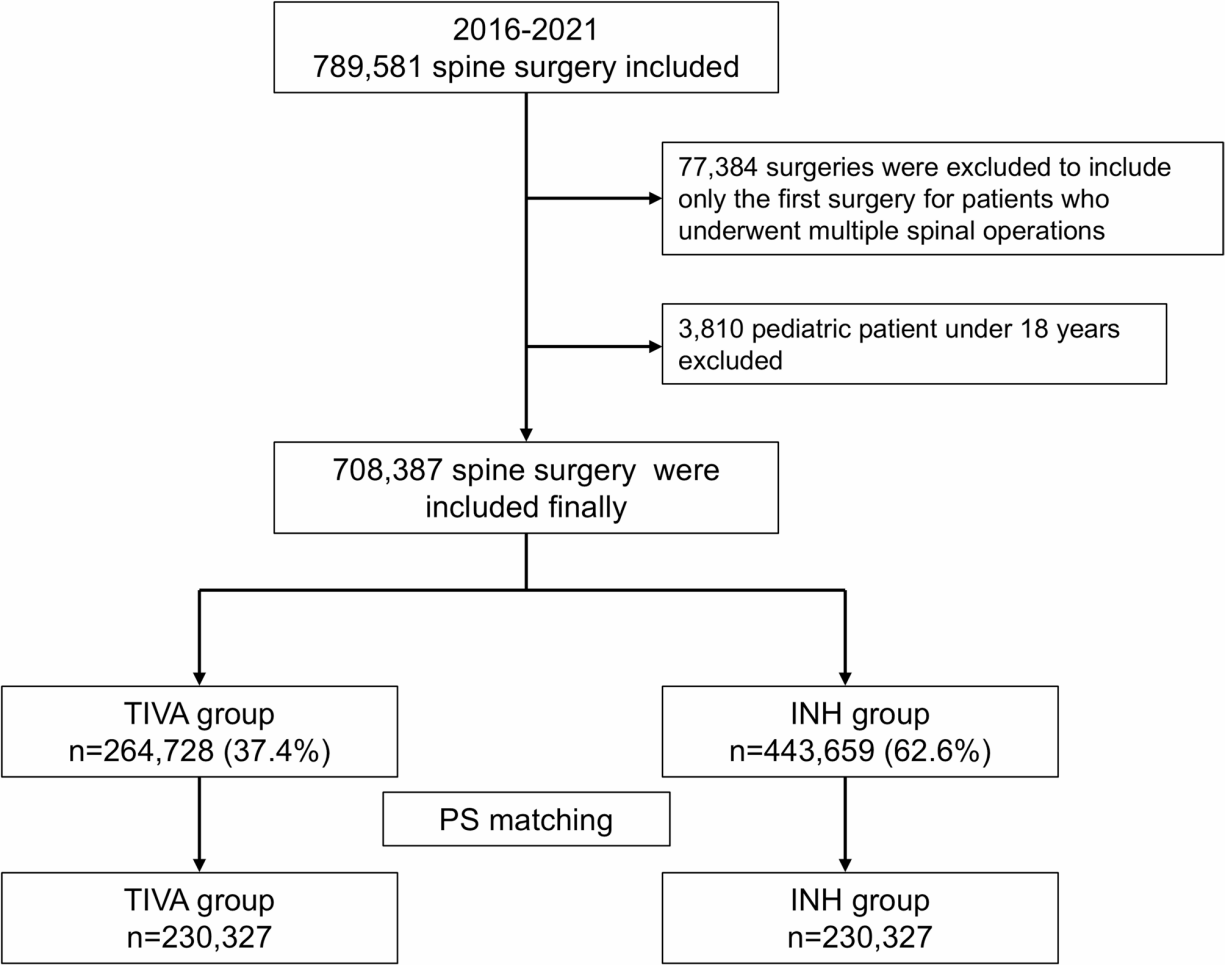
Flowchart depicting the patient selection process PS, propensity score; TIVA, total intravenous anesthesia; INH, inhalation

Figure 1 illustrates the patient selection process. Among 789,581 spinal surgery cases between 2016 and 2021, 708,387 satisfied the inclusion criteria. The patients were divided into two groups based on the type of anesthesia used: 264,728 patients (37.4%) in the TIVA group and 443,659 patients (62.6%) in the INH group. Following PS matching, 460,654 patients (230,327 in each group) were included in the analysis. Table 1 compares the clinicopathological features of the TIVA and INH groups before and after PS matching. The ASDs of variables between the two groups were < 0.1, indicating good balance after PS matching.

**Table 1:** Characteristics of the TIVA and INH group before and after PS matching

Variable		Before PS matching (*n* = 708,387)		ASD	After PS matching (*n* = 460,654)		ASD
		TIVA *n* = 264,728	INH *n* = 443,659		TIVA *n* = 230,327	INH *n* = 230,327	
Age, year		61.4 (14.4)	62.4 (13.7)	0.076	61.7 (14.0)	61.6 (14.3)	0.008
Sex, male		136,817 (51.7)	225,012 (50.7)	0.018	121,005 (52.5)	117,872 (51.2)	0.025
Having a job		171,270 (64.7)	280,297 (63.2)	0.024	148,985 (64.7)	148,991 (64.7)	0.002
Residence at surgery							
	Urban area	103,423 (39.1)	172,101 (38.8)		90,600 (39.3)	89,900 (39.0)	
	Rural area	161,305 (60.9)	271,558 (61.2)	0.038	139,727 (60.7)	140,427 (61.0)	0.016
Household income level							
	Q1 (lowest)	45,593 (17.2)	76,987 (17.4)		39,843 (17.3)	39,589 (17.3)	
	Q2	48,022 (18.1)	79,094 (17.8)	0.001	41,869 (18.2)	41,752 (18.1)	0.002
	Q3	65,154 (24.6)	106,467 (24.0)	0.008	56,946 (24.7)	56,901 (24.7)	0.002
	Q4 (highest)	89,912 (34.0)	148,834 (33.5)	0.008	77,546 (33.7)	77,996 (33.9)	0.002
	Medical aid program group	11,612 (4.4)	25,216 (5.7)	0.044	10,240 (4.4)	10,027 (4.4)	< 0.001
	Unknown	4,435 (1.7)	7,061 (1.6)	0.002	3,883 (1.7)	3,792 (1.6)	0.005
CCI		0.9 (1.3)	1.1 (1.4)	0.028	0.9 (1.3)	0.9 (1.2)	0.021
	Myocardial infarction	4,413 (1.7)	6,958 (1.6)	0.010	4.104 (1.8)	3,855 (1.7)	0.009
	Congestive heart failure	4,735 (1.8)	15,307 (3.5)	0.132	4,373 (1.9)	4,238 (1.8)	0.004
	Peripheral vascular disease	1,895 (0.7)	4,127 (0.9)	0.025	1,694 (0.7)	1,672 (0.7)	0.001
	Cerebrovascular disease	6,261 (2.4)	17,066 (3.8)	0.102	5,670 (2.5)	5,421 (2.4)	0.009
	Dementia	2,100 (0.8)	6,931 (1.6)	0.075	1,957 (0.8)	1,840 (0.8)	0.004
	Chronic pulmonary disease	21,658 (8.2)	64,335 (14.5)	0.227	19,894 (8.6)	17,821 (7.7)	0.031
	Rheumatic disease	8,779 (3.3)	15,640 (3.5)	0.015	8,297 (3.6)	7,830 (3.4)	0.016
	Peptic ulcer disease	26,928 (10.2)	49,607 (11.2)	0.032	23,656 (10.3)	23,543 (10.2)	0.001
	Mild liver disease	49,563 (18.7)	99,592 (22.4)	0.118	44,194 (19.2)	46,247 (20.1)	0.024
	DM without chronic complication	56,907 (21.5)	105,283 (23.7)	0.044	50,609 (22.0)	50,656 (22.0)	0.002
	DM with chronic complication	5,046 (1.9)	13,113 (3.0)	0.080	4,550 (2.0)	4,396 (1.9)	0.007
	Hemiplegia or paraplegia	6,884 (2.6)	15,688 (3.5)	0.077	5,887 (2.6)	5,813 (2.5)	0.014
	Renal disease	2,557 (1.0)	6,918 (1.6)	0.064	2,333 (1.0)	2,238 (1.0)	0.003
	Cancer	4,904 (1.9)	10,303 (2.3)	0.038	3,838 (1.7)	3,333 (1.4)	0.018
	Moderate and severe liver disease	190 (0.1)	683 (0.2)	0.028	177 (0.1)	177 (0.1)	0.002
	Metastatic cancer	2,390 (0.9)	3,633 (0.8)	0.014	1,650 (0.7)	1,332 (0.6)	0.013
	AIDS/HIV	46 (0.0)	2265 (0.1)	0.028	42 (0.0)	41 (0.0)	0.002
Underlying disability							
	Mild to moderate disability	31,318 (11.8)	64,040 (14.4)	0.075	27,639 (12.0)	26,854 (11.7)	0.007
	Severe disability	7,404 (2.8)	14,599 (3.3)	0.025	6,217 (2.7)	6,158 (2.7)	0.005
IONM		22,415 (8.5)	6,447 (1.5)		12,198 (5.3)	6,399 (2.8)	0.081
Type of spine surgery							
	Arthrodesis	22,633 (8.5)	67,112 (15.1)		19,722 (8.6)	15,956 (6.9)	
	Corpectomy	329 (0.1)	457 (0.1)	0.008	231 (0.1)	191 (0.1)	0.004
	Spine fracture surgery	4,322 (1.6)	3,166 (0.7)	0.072	2,466 (1.1)	2,844 (1.2)	0.013
	Discectomy	145,342 (54.9)	244,899 (55.2)	0.025	125,414 (54.5)	135,414 (58.8)	0.081
	Spine tumor surgery	5,681 (2.1)	4,743 (1.1)	0.085	3,214 (1.4)	2,384 (1.0)	0.021
	Laminectomy	76,520 (28.9)	110,576 (24.9)	0.082	72,204 (31.3)	67,681 (29.4)	0.039
	Other spine surgery	9,901 (3.7)	12,706 (2.9)	0.038	7,076 (3.1)	5,857 (2.5)	0.029
Type of surgical spine level							
	Cervical	24,888 (9.4)	65,130 (14.7)		20,294 (8.8)	15,603 (6.8)	
	Thoracic	8,810 (3.3)	16,931 (3.8)	0.001	6,233 (2.7)	4,842 (2.1)	0.035
	Lumbar	220,444 (83.3)	354,694 (79.9)	0.054	197,237 (85.6)	203,529 (88.4)	0.068
	Not specific	10,586 (4.0)	6,904 (1.6)	0.119	6,563 (2.8)	6,353 (2.8)	0.009
Year of surgery							
	2016	70,820 (26.8)	47,882 (10.8)	0.349	46,637 (20.2)	43,840 (19.0)	0.029
	2017	37,088 (14.0)	79,374 (17.9)	0.113	35,433 (15.4)	36,305 (15.8)	0.009
	2018	37,397 (14.1)	77,647 (17.5)	0.091	35,590 (15.5)	36,108 (15.7)	0.010
	2019	38,889 (14.7)	76,895 (17.3)	0.072	36,681 (15.9)	37,139 (16.1)	0.002
	2020	39,633 (15.0)	77,667 (17.5)	0.065	37,296 (16.2)	37,585 (16.3)	0.005
	2021	40,901 (15.5)	84,194 (19.0)	0.095	38,690 (16.8)	39,350 (17.1)	0.009

*TIVA *total intravenous anaesthesia, *INH *inhalation, *PS* propensity score, *ASD* absolute standardized differences, *CCI* Charlson comorbidity index, *DM* diabetes mellitus, *HIV* human immunodeficiency virus, *AIDS* Acquired immunodeficiency syndrome, *IONM* intraoperative neurophysiological monitoring

### Analyses in the PS-matched cohort

Table 2 presents the results of the analyses in the PS-matched cohort. In this cohort, the in-hospital mortality rate in the TIVA group was 0.10% (229/230,327), whereas the rate in the INH group was 0.13% (292/230,327). Univariable logistic regression in the PS‑matched cohort showed that the TIVA group had lower odds of in‑hospital mortality compared to the INH group (OR 0.85; 95% CI 0.80–0.89; *P* = 0.004).The incidence of postoperative complications in the TIVA group was 11.8% (27,217/230,327), whereas the incidence in the INH group was 14.2% (32,730/230,327). Univariable logistic regression in the PS‑matched cohort showed that the TIVA group had lower odds of postoperative complications compared to the INH group (OR: 0.81, 95% CI: 0.80, 0.82; *P* < 0.001). A detailed breakdown of key complications revealed that TIVA was associated with significantly lower odds of pulmonary embolism (OR: 0.85, 95% CI: 0.78, 0.92; *P* < 0.001), acute and subacute hepatic failure (OR: 0.48, 95% CI: 0.31, 0.73; *P* = 0.001), acute kidney injury (OR: 0.64, 95% CI: 0.57, 0.72; *P* < 0.001), sepsis (OR: 0.49, 95% CI: 0.45, 0.54; *P* < 0.001), wound infection (OR: 0.61, 95% CI: 0.59, 0.64; *P* < 0.001), pneumonia (OR: 0.91, 95% CI: 0.86, 0.96; *P* = 0.001), urinary tract infection (OR: 0.88, 95% CI: 0.84, 0.91; *P* < 0.001), central nervous system complication (OR: 0.75, 95% CI: 0.71, 0.79; *P* < 0.001), and hemorrhage (OR: 0.85, 95% CI: 0.82, 0.87; *P* < 0.001).

**Table 2:** Results of analyses in the PS-matched cohort

Outcome		Event (%)	OR (95% CI)	*P*-value
In-hospital mortality				
	Inhalation group	292/230,327 (0.13)	1	
	TIVA group	229/230,327 (0.10)	0.85 (0.80, 0.89)	0.004
Postoperative complication				
	Inhalation group	32,730/230,327 (14.2)	1	
	TIVA group	27,217/230,327 (11.8)	0.81 (0.80, 0.82)	< 0.001
Acute coronary events				
	Inhalation group	3,855/230,327 (1.7)	1	
	TIVA group	4,104/230,327 (1.8)	1.07 (1.02, 1.11)	0.005
Cerebral infarction or hemorrhage				
	Inhalation group	1,950/230,327 (0.8)	1	
	TIVA group	2,064/230,327 (0.9)	1.06 (0.99, 1.13)	0.071
Heart failure				
	Inhalation group	3,594/230,327 (1.6)	1	
	TIVA group	3,774/230,327 (1.6)	1.05 (1.00, 1.10)	0.065
Pulmonary embolism				
	Inhalation group	1,187/230,327 (0.5)	1	
	TIVA group	1,006/230,327 (0.4)	0.85 (0.78, 0.92)	< 0.001
Acute and subacute hepatic failure				
	Inhalation group	67/230,327 (0.0)	1	
	TIVA group	32/230,327 (0.0)	0.48 (0.31, 0.73)	0.001
Acute kidney injury				
	Inhalation group	743/230,327 (0.3)	1	
	TIVA group	476/230,327 (0.2)	0.64 (0.57, 0.72)	< 0.001
Sepsis				
	Inhalation group	1,378/230,327 (0.6)	1	
	TIVA group	679/230,327 (0.3)	0.49 (0.45, 0.54)	< 0.001
Wound infection				
	Inhalation group	6,534/230,327 (2.8)	1	
	TIVA group	4,036/230,327 (1.8)	0.61 (0.59, 0.64)	< 0.001
Pneumonia				
	Inhalation group	2,602/230,327 (1.1)	1	
	TIVA group	2,360/230,327 (1.0)	0.91 (0.86, 0.96)	0.001
Urinary tract infection				
	Inhalation group	5,135/230,327 (2.2)	1	
	TIVA group	4,512/230,327 (2.0)	0.88 (0.84, 0.91)	< 0.001
CNS complication				
	Inhalation group	3,261/230,327 (1.4)	1	
	TIVA group	2,439/230,327 (1.1)	0.75 (0.71, 0.79)	< 0.001
Hemorrhage				
	Inhalation group	9,823/230,327 (4.3)	1	
	TIVA group	8,367/230,327 (3.6)	0.85 (0.82, 0.87)	< 0.001

*PS* propensity score, *OR* odds ratio, *CI* confidence interval, *TIVA* total intravenous anesthesia, *CNS* central nervous system

### Analyses in the entire cohort

To ensure that our findings were not an artifact of the matching process or reduced sample size, we conducted multivariable logistic regression in the full cohort of 708,387 patients, adjusting for the same prespecified covariates used in matching: age, sex, household income quartile, residence (urban/rural), employment status, Charlson Comorbidity Index, disability level, spinal level (cervical, thoracic, lumbar), procedure type (arthrodesis, corpectomy, fracture repair, discectomy, tumor surgery, laminectomy, other), and IONM use.

As shown in Table 3, Model 1 (in‑hospital mortality) confirmed that TIVA was associated with 26% lower odds of death compared to INH (OR 0.74; 95% CI 0.63–0.87; *P* < 0.001). Model 2 (postoperative complications) similarly demonstrated 29% lower odds of complications in the TIVA group (OR 0.71; 95% CI 0.70–0.73; *P* < 0.001).

**Table 3:** Multivariable logistic regression model outcomes for postoperative complications of spinal surgery

Variable		OR (95% CI)	*P*-value
In-hospital mortality (model 1), (TIVA vs. INH)		0.74 (0.63, 0.87)	< 0.001
Postoperative complication (model 2), (TIVA vs. INH)		0.71 (0.70, 0.73)	< 0.001
	Acute coronary events, (TIVA vs. INH)	1.41 (1.36, 1.47)	< 0.001
	Cerebral infarction or hemorrhage, (TIVA vs. INH)	0.72 (0.68, 0.75)	< 0.001
	Heart failure, (TIVA vs. INH)	0.64 (0.61, 0.66)	< 0.001
	Pulmonary embolism, (TIVA vs. INH)	0.67 (0.62, 0.72)	< 0.001
	Acute and subacute hepatic failure, (TIVA vs. INH)	0.42 (0.29, 0.61)	< 0.001
	Acute kidney injury, (TIVA vs. INH)	0.48 (0.44, 0.54)	< 0.001
	Sepsis, (TIVA vs. INH)	0.37 (0.34, 0.41)	< 0.001
	Wound infection, (TIVA vs. INH)	0.59 (0.57, 0.61)	< 0.001
	Pneumonia, (TIVA vs. INH)	0.67 (0.64, 0.71)	< 0.001
	Urinary tract infection, (TIVA vs. INH)	0.79 (0.77, 0.82)	< 0.001
	CNS complication, (TIVA vs. INH)	0.66 (0.63, 0.69)	< 0.001
	Hemorrhage, (TIVA vs. INH)	0.84 (0.81, 0.86)	< 0.001

*OR* odds ratio, *CI* confidence interval, *TIVA* total intravenous anaesthesia, *INH* inhalation, *CNS* central nervous system

Independent predictors of increased in‑hospital mortality included older age, male sex, higher Charlson Comorbidity Index score, and preexisting severe disability. Conversely, IONM use was independently associated with reduced mortality, likely reflecting propofol’s superior compatibility with neurophysiological monitoring. Additionally, surgical factors such as corpectomy, thoracic spine level, and non‑laminectomy procedures were linked to higher mortality risk, whereas fracture repair, discectomy, laminectomy, and lumbar‑level surgery were associated with lower risk.

### Subgroup analyses

Table 4 presents subgroup analyses based on the surgical spine level. For patients who had surgery at the thoracic spine, those who received TIVA had a lower association with in-hospital mortality (OR: 0.71, 95% CI: 0.80, 0.99; *P* = 0.048) and postoperative complications (OR: 0.80, 95% CI: 0.73, 0.87; *P* < 0.001) compared with those who received volatile anaesthetics. Similarly, for patients who underwent surgery at the lumbar level, those who received TIVA were less associated with in-hospital mortality (OR: 0.42, 95% CI: 0.30, 0.57; *P* < 0.001) and postoperative complications (OR 0.75, 95% CI: 0.74, 0.77; *P* < 0.001) compared with those who received inhalation anaesthetics.

**Table 4:** Subgroup analysis

Variable		OR (95% CI)	*P*-value
Cervical spine surgery			
	In-hospital mortality, (TIVA vs. INH)	1.15 (0.80, 1.67)	0.475
	Postoperative complication, (TIVA vs. INH)	0.92 (0.87, 0.98)	0.005
Thoracic spine surgery			
	In-hospital mortality, (TIVA vs. INH)	0.71 (0.80, 0.99)	0.048
	Postoperative complication, (TIVA vs. INH)	0.80 (0.73, 0.87)	< 0.001
Lumbar spine surgery			
	In-hospital mortality, (TIVA vs. INH)	0.42 (0.30, 0.57)	< 0.001
	Postoperative complication, (TIVA vs. INH)	0.75 (0.74, 0.77)	< 0.001
Not specific level spine surgery			
	In-hospital mortality, (TIVA vs. INH)	1.02 (0.68, 1.54)	0.925
	Postoperative complication, (TIVA vs. INH)	0.63 (0.57, 0.70)	< 0.001

*TIVA* total intravenous anaesthesia, *INH* inhalation

## Discussion

### Summary of key findings

In our retrospective study of 708,387 adult patients who underwent spinal surgery, we observed that TIVA was associated with lower in-hospital mortality and fewer postoperative complications than volatile inhalants. This trend was consistent across the thoracic and lumbar levels.

### Comparison with prior literature

Our study underscores that using TIVA correlates with lower in-hospital mortality than using volatile inhalants. The effect of anaesthetic methods on mortality is a topic of ongoing debate. For instance, mortality is approximately 50% greater with volatile anesthesia than with intravenous anesthesia in patients undergoing cancer surgery [9]. Although volatile anaesthetics have been linked to reduced mortality in cardiac surgery, the results are inconsistent with those of patients undergoing non-cardiac surgery [8]. A meta-analysis by Schraag et al. (2018) found that patients undergoing general surgeries showed no difference in in-hospital mortality between the TIVA and volatile anaesthetics groups. However, these results can vary depending on patient selection or the type of surgery. Hsu et al. observed that the TIVA group had a shorter hospital stay than the inhalants group [7]. This decreased the chance of in-hospital mortality and reduced the risk of opportunistic infections and other adverse events.

### Mechanistic and technical considerations

TIVA results in fewer postoperative complications than volatile inhalants. Propofol, a key component of TIVA, exhibits antioxidant properties by directly scavenging reactive oxygen species, forming propofol‑derived phenoxyl radicals, and enhancing endogenous antioxidant defenses [4, 16]. Beyond its antioxidant effects, propofol also modulates the surgical inflammatory response: in a prospective randomized trial of robot‑assisted laparoscopic prostatectomy, propofol‑based TIVA significantly lowered perioperative interleukin (IL)‑6 and tumor necrosis factor (TNF)‑α levels compared with sevoflurane [17], although a 2021 meta‑analysis of 23 RCTs found no consistent differences in IL‑6, IL‑10, or TNF‑α between propofol and volatile agents [18]. Clinically, this pharmacologic profile may underlie the reductions in inflammatory complications we observed—such as acute kidney injury, sepsis, wound infection, and urinary tract infection—as supported by Yoo et al.’s finding that TIVA reduced both the incidence and severity of acute kidney injury after valvular heart surgery compared with sevoflurane [19]. Clarifying how propofol’s antioxidant and immunomodulatory actions translate into better outcomes after spinal surgery will require dedicated mechanistic and clinical studies.

IONM is commonly employed in spinal surgeries where there is a potential for neurological injury [20]. The immediate identification of impaired nerve function through IONM provides the surgeon with an opportunity to address potential issues and prevent lasting damage [20]. The findings of our study indicate that the proportion of IONM used during spine surgery was notably higher in the TIVA group than in the INH group. This is attributed to the established understanding that IONM frequently counteracts the effects of muscle relaxants, whereas TIVA is considered more suitable for sustaining anesthesia [21]. While IONM use was adjusted for in our multivariable models, its higher frequency in the TIVA group may reflect greater case complexity; future studies should consider stratified or instrumental‑variable approaches to isolate the independent effect of IONM.

### Limitations and future directions

However, this study has some limitations. First, the NHIS claims database is based on prescription and diagnostic coding and does not include intraoperative anesthetic depth monitoring (e.g., bispectral index values) or specific dosing metrics (e.g., minimum alveolar concentration for volatile agents or total propofol dosage), precluding dose–response or depth‑of‑anesthesia analyses. Although existing evidence suggests that anesthetic depth and dosing do not significantly alter mortality or complication rates after major surgery [22], a dose-dependent relationship may exist between using anaesthetics and adverse outcomes risks. Second, details on operative complexity—such as operative duration, estimated blood loss, and number of spinal levels operated—were not available, limiting our ability to adjust for surgical complexity. Post‑discharge outcomes (e.g., 30‑ and 90‑day complications) could not be captured; future registry or claims‑linkage studies are needed. Third, subgroup sample sizes may limit statistical power, and dedicated biomarker studies are required to validate propofol’s proposed anti‑inflammatory mechanisms. Fourth, approximately 10% of procedures represented subsequent spinal operations and were excluded to preserve independence of observations. The distribution of these excluded repeat surgeries by anesthesia type could potentially influence our findings; because our NHIS data access period has ended, we were unable to re-tabulate excluded-case anesthesia distributions or include a ‘prior spinal surgery’ indicator in additional adjusted models. If data access is restored we will perform these sensitivity analyses and report results. Finally, without well-designed large multicentre randomised controlled trials, there is insufficient information to debate the superiority of one anaesthetic approach over another in terms of in-hospital mortality and early postoperative complications. We await the results from the Trajectories of Recovery after Intravenous Propofol versus Inhaled Volatile Anesthesia (THRIVE) trial funded by the Patient-Centered Outcomes Research Institute in the United States [23] and the Volatile vs. Total Intravenous Anesthesia for Major Non-cardiac Surgery (VITALS) trial funded by the National Institute for Health Research in the United Kingdom [24]. However, these trials do not specifically target patients undergoing spinal surgery.

## Conclusions

In conclusion, our nationwide population-based study found that TIVA was associated with improved in-hospital mortality risk and fewer postoperative complications following spinal surgery compared with volatile inhalants. This suggests that TIVA can be a superior option for anesthesia in spinal surgery. Further research is necessary to develop a more personalised anesthesia technique that considers each patient’s specific diseases and surgical procedures.

## Supplementary Information

Supplementary material 1.

Supplementary material 2.

Supplementary material 3.

Supplementary material 4.

## Data Availability

Data are available from the corresponding author upon reasonable request.

## Abbreviations

TIVA: Total intravenous anesthesia
INH: Inhalation anesthesia
PS: Propensity score
NHIS: National health insurance service
ICD-10: International classification of diseases, tenth revision
OR: Odds ratio
CI: Confidence interval
ASD: Absolute standardised difference
IONM: Intraoperative neurophysiological monitoring
THRIVE: Trajectories of recovery after intravenous propofol versus inhaled volatile anesthesia
VITALS: Volatile vs. total intravenous anesthesia for major non-cardiac surgery
