# Supplementary material for: Propofol TIVA vs. inhalational anesthesia for spine surgery: in‑hospital mortality and postoperative complications in a nationwide Korean cohort
Source: BMC Anesthesiol. 2025 Oct 17;25:508. doi: 10.1186/s12871-025-03385-4 (PMC12533432; doi:10.1186/s12871-025-03385-4)
Supplement: Supplementary file 1 — Supplementary material 1. [file 12871_2025_3385_MOESM1_ESM.docx]

Table S1. Code of spine surgery in this study

| **Op code** | **Op name** |
| --- | --- |
| M5952 | Emergency Spine Immobilization |
| N0303 | Osteotomy[Spine,Pelvis] |
| N0444 | Arthrodesis For Spinal Deformity(Anterior Technique)-7 Level Below |
| N0445 | Arthrodesis For Spinal Deformity(Anterior Technique)-7 Level Over |
| N0446 | Arthrodesis For Spinal Deformity(Posterior Technique)-7 Level Below |
| N0447 | Arthrodesis For Spinal Deformity(Posterior Technique)-7 Level Over |
| N0451 | Vertebral Corpectomy(Cervical Spine) |
| N0452 | Vertebral Corpectomy(Thoracic Spine) |
| N0453 | Vertebral Corpectomy(Lumbar Spine) |
| N0454 | Surgical removal of the ossification of spinal ligament(OPLL removal-anterior approach) |
| N0455 | Surgical removal of the ossification of spinal ligament(OLF removal-posterior approach) |
| N0466 | Arthrodesis of Spine-Lumbar Spine-Anterior Technique |
| N0468 | Arthrodesis of Spine-Thoracic Spine-Posterior Technique |
| N0469 | Arthrodesis of Spine-Lumbar Spine-Posterior Technique |
| N0480 | Operation of Spina Bifida |
| N0591 | Open Reduction of Fracture and Dislocation of Spine or Pelvis-Spine |
| N0592 | Open Reduction of Fracture and Dislocation of Spine or Pelvis-Acetabulum |
| N0593 | Open Reduction of Fracture and Dislocation of Spine or Pelvis-Pelvis |
| N0630 | Closed Reduction of Fracture And/Or Dislocated Spine |
| N1466 | Arthrodesis of Spine-Lumbar Spine-Anterior Technique |
| N1469 | Arthrodesis of Spine-Lumbar Spine-Posterior Technique |
| N1491 | Diskectomy(Invasive)-Cervical Spine |
| N1492 | Diskectomy(Invasive)-Thoracic Spine |
| N1493 | Diskectomy(Invasive)-Lumbar Spine |
| N1498 | Laminectomy, Thoracic Spine |
| N1499 | Laminectomy, Lumbar Spine |
| N2461 | Arthrodesis of Spine-Cervical Spine-Anterior Technique[Trans-Oral] |
| N2462 | Arthrodesis of Spine-Cervical Spine-Anterior Technique[Anterior Odontoid Screw Fixation] |
| N2463 | Arthrodesis of Spine-Cervical Spine-Anterior Technique[Others] |
| N2464 | Arthrodesis of Spine-Thoracic Spine-Anterior Technique[Transmanubrial] |
| N2465 | Arthrodesis of Spine-Thoracic Spine-Anterior Technique[Transsternal] |
| N2466 | Arthrodesis of Spine-Thoracic Spine-Anterior Technique[Others] |
| N2467 | Arthrodesis of Spine-Cervical Spine-Posterior Technique[Occipito-Cervical Fusion] |
| N2468 | Arthrodesis of Spine-Cervical Spine-Posterior Technique[C1-2 Fixation] |
| N2469 | Arthrodesis of Spine-Cervical Spine-Posterior Technique[Others] |
| N2471 | Removal of Implant For Internal Fixation of Spine[Anterior] |
| N2472 | Removal of Implant For Internal Fixation of Spine[Posterior] |
| N2491 | Cervical Spine Laminoplasty |
| N2492 | Cervical Spine Laminoplasty |
| N2497 | Laminectomy, Cervical Spine |
| N2498 | Laminectomy, Thoracic Spine |
| N2499 | Laminectomy, Lumbar Spine |
| S4671 | Operation of Spinal Arteriovenous Malformation |
| S4685 | Operation of CNS Anomaly-Tethered Spinal Cord |
| S4694 | Excision of Intramedullary Tumor Or Lesion-Cervical Spine |
| S4695 | Excision of Intramedullary Tumor Or Lesion-Thoracic Spine |
| S4696 | Excision of Intramedullary Tumor Or Lesion-Lumbar Spine |
| S4704 | Excision of Extradural Tumor Or Lesion-Cervical Spine-Involving Pedicle And/Or Vertebral Body |
| S4705 | Excision of Extradural Tumor Or Lesion-Cervical Spine-Without Pedicle And Vertebral Body |
| S4706 | Excision of Extradural Tumor Or Lesion-Thoracic Spine-Involving Pedicle And/Or Vertebral Body |
| S4707 | Excision of Extradural Tumor Or Lesion-Thoracic Spine-Without Pedicle And Vertebral Body |
| S4708 | Excision of Extradural Tumor Or Lesion-Lumbar Spine-Involving Pedicle And/Or Vertebral Body |
| S4709 | Excision of Extradural Tumor Or Lesion-Lumbar Spine-Without Pedicle And Vertebral Body |
| S6691 | Excision of Intradural Tumor Or Lesion-Cervical Spine |
| S6692 | Excision of Intradural Tumor Or Lesion-Cervical Spine |
| S6693 | Excision of Intradural Tumor Or Lesion-Thoracic Spine |
| S6694 | Excision of Intradural Tumor Or Lesion-Thoracic Spine |
