# Supplementary material for: Propofol TIVA vs. inhalational anesthesia for spine surgery: in‑hospital mortality and postoperative complications in a nationwide Korean cohort
Source: BMC Anesthesiol. 2025 Oct 17;25:508. doi: 10.1186/s12871-025-03385-4 (PMC12533432; doi:10.1186/s12871-025-03385-4)
Supplement: Supplementary file 4 — Supplementary material 4. [file 12871_2025_3385_MOESM4_ESM.docx]

Table S4. All ORs with 95% CIS in other covariates in model 1

| Variable | | OR (95% CI) | *P*-value |
| --- | --- | --- | --- |
| Age, year | | 1.04 (1.03, 1.05) | <0.001 |
| Sex, male | | 1.62 (1.40, 1.87) | <0.001 |
| Having a job | | 0.88 (0.76, 1.02) | 0.098 |
| Residence at surgery | |  |  |
|  | Urban area | 1 |  |
|  | Rural area | 0.90 (0.79, 1.03) | 0.123 |
| Household income level | |  |  |
|  | Q1 (lowest) | 1 |  |
|  | Q2 | 1.21 (0.96, 1.53) | 0.113 |
|  | Q3 | 1.05 (0.84, 1.32) | 0.672 |
|  | Q4 (highest) | 1.11 (0.90, 1.37) | 0.321 |
|  | Medical aid program group | 1.27 (0.95, 1.69) | 0.109 |
|  | Unknown | 1.23 (0.72, 2.13) | 0.449 |
| CCI | | 1.43 (1.40, 1.46) | <0.001 |
|  | Myocardial infarction | 2.11 (1.52, 2.91) | <0.001 |
|  | Congestive heart failure | 3.51 (2.93, 4.19) | <0.001 |
|  | Peripheral vascular disease | 1.75 (1.20, 2.56) | 0.004 |
|  | Cerebrovascular disease | 2.11 (1.73, 2.57) | <0.001 |
|  | Dementia | 0.70 (0.47, 1.04) | 0.076 |
|  | Chronic pulmonary disease | 1.90 (1.64, 2.21) | <0.001 |
|  | Rheumatic disease | 0.56 (0.36, 0.86) | 0.009 |
|  | Peptic ulcer disease | 0.89 (0.73, 1.10) | 0.298 |
|  | Mild liver disease | 0.94 (0.80, 1.12) | 0.500 |
|  | DM without chronic complication | 1.53 (1.33, 1.77) | <0.001 |
|  | DM with chronic complication | 1.26 (0.93, 1.72) | 0.143 |
|  | Hemiplegia or paraplegia | 1.57 (1.26, 1.95) | <0.001 |
|  | Renal disease | 3.10 (2.43, 3.96) | <0.001 |
|  | Cancer | 2.52 (1.97, 3.23) | <0.001 |
|  | Moderate and severe liver disease | 8.60 (5.69, 13.0) | <0.001 |
|  | Metastatic cancer | 5.13 (3.87, 6.82) | <0.001 |
|  | AIDS/HIV | 2.66 (0.64, 11.06) | 0.178 |
| Underlying disability | |  |  |
|  | Mild to moderate disability | 0.77 (0.50, 1.21) | 0.265 |
|  | Severe disability | 2.11 (1.71, 2.61) | <0.001 |
| IONM | | 0.73 (0.57, 0.92) | 0.008 |
| Type of spine surgery | |  |  |
|  | Arthrodesis | 1 |  |
|  | Corpectomy | 3.44 (2.01, 5.91) | <0.001 |
|  | Spine fracture | 0.21 (0.12, 0.37) | <0.001 |
|  | Discectomy | 0.30 (0.23, 0.39) | <0.001 |
|  | Spine tumor | 1.19 (0.93, 1.52) | 0.175 |
|  | Laminectomy | 0.69 (0.56, 0.87) | 0.001 |
|  | Other spine surgery | 0.78 (0.56, 1.07) | 0.125 |
| Type of surgical spine level | |  |  |
|  | Cervical | 1 |  |
|  | Thoracic | 1.26 (1.00, 1.58) | 0.046 |
|  | Lumbar | 0.48 (0.38, 0.61) | <0.001 |
|  | Not specific | 6.04 (4.28, 8.54) | <0.001 |
| Year of surgery | |  |  |
|  | 2016 | 1 |  |
|  | 2017 | 1.07 (0.84, 1.36) | 0.591 |
|  | 2018 | 0.85 (0.67, 1.09) | 0.211 |
|  | 2019 | 0.93 (0.73, 1.18) | 0.525 |
|  | 2020 | 0.99 (0.78, 1.25) | 0.914 |
|  | 2021 | 0.87 (0.69, 1.10) | 0.246 |

OR, odds ratio; CI, confidence interval; CCI, Charlson comorbidity index; DM, diabetes mellitus; HIV, human immunodeficiency virus; AIDS, Acquired immunodeficiency syndrome; IONM, intraoperative neurophysiological monitoring
